# Heat Tolerance and Its Plasticity in Freshwater and Marine Fishes Reflect Exposure to Extremes and Seasonal Variation in Habitat Temperatures

**DOI:** 10.1111/ele.70341

**Published:** 2026-03-04

**Authors:** Wilco C. E. P. Verberk, Erin Henry, Félix P. Leiva, Valerio Barbarossa, Aafke M. Schipper

**Affiliations:** ^1^ Department of Ecology, RIBES Radboud University Nijmegen the Netherlands; ^2^ Wildlife Ecology and Conservation Group Wageningen University and Research Wageningen the Netherlands; ^3^ Integrative Ecophysiology Alfred Wegener Institute Helmholtz Centre Polar and Marine Research Bremerhaven Germany; ^4^ Department of Environmental Science, RIBES Radboud University Nijmegen the Netherlands; ^5^ Institute of Environmental Sciences Leiden University Leiden the Netherlands; ^6^ PBL Netherlands Environmental Assessment Agency The Hague the Netherlands

**Keywords:** acclimation, habitat temperature, latitude, macrophysiology, thermal tolerance

## Abstract

Large‐scale investigations of patterns in heat tolerance often use latitude as a proxy for thermal conditions. We examined how heat tolerance of fishes and its plasticity relate to thermal extremes and seasonal fluctuations in habitat temperatures. Our dataset included over 3000 heat tolerance measurements from 500+ fish species inhabiting freshwater, marine and brackish environments. While heat tolerance varied with latitude, extremes and seasonal fluctuations in temperature better explained this variation. We also found that freshwater fishes had higher heat tolerance with greater plasticity than marine fishes, reflecting exposure to higher and more variable temperatures. Plasticity declined as they neared their thermal limits, highlighting the challenge of physiologically adjusting to extreme heat stress. Finally, we found that the benefits of plasticity in terms of time gained for adaptation varied considerably across the globe (5%–35%), with the lowest values for marine species at lower (warmer) latitudes. The distinct heat tolerance adaptations in freshwater and marine fishes reported here underscore the importance of thermal physiology in predicting responses to climate change.

## Graphical abstract

We found that freshwater fishes had higher heat tolerance with greater plasticity than marine fishes, reflecting exposure to higher and more variable temperatures. Accordingly, the benefits of plasticity in terms of time gained for adaptation varied considerably across the globe (5%–35%), with the lowest values for marine species at lower (warmer) latitudes. The distinct heat tolerance adaptations in freshwater and marine fishes reported here underscore the importance of thermal physiology in predicting responses to climate change.

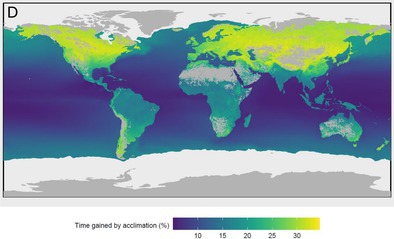

## 1 Introduction

Earth's climate is warming, exposing organisms to increasingly higher and more variable temperatures (IPCC 2018). Biological responses to warming include poleward range shifts and changes in phenology, which help organisms to track their thermal niches (Lenoir et al. 2020; Sunday et al. 2012). Understanding the vulnerability of species to climate warming requires information on the thermal extremes to which they are exposed in their habitats as well as their sensitivity to heat. Large‐scale syntheses typically report strong latitudinal variation in both exposure to heat and the sensitivity of species to heat, which is frequently measured as their heat tolerance. In general, and not surprisingly, tropical species are better able to tolerate heat while also being more exposed to heat (Deutsch et al. 2008; Pinsky et al. 2019; Pörtner and Peck 2010).

Many fish species are able to modulate their heat tolerance following acclimation to warmer temperatures (Brett 1971; Comte and Olden 2017b; McKenzie et al. 2021). Such plasticity in heat tolerance can be quantified by the acclimation response ratio (ARR), which expresses the gain in heat tolerance for every degree of warm acclimation (Claussen 1977; Stillman 2003). Thus, a species with an ARR of 0 would exhibit no plasticity in heat tolerance, while a species with an ARR of 1 would be able to perfectly acclimate and, if allowed to acclimate, never suffer from heat mortality. In reality, the values of ARR are below 1, and so plasticity in heat tolerance has been argued to have limited potential to buffer ectotherms from global warming (Gunderson and Stillman 2015; Barley et al. 2021), although overheating can be substantially delayed by such plasticity, providing additional time for evolutionary adaptation (Morley et al. 2019).

Basal heat tolerance is expected to be highest at low, tropical latitudes where heat exposure is greatest, while plasticity in heat tolerance may peak at temperate latitudes where thermal variability is greatest (Bozinovic et al. 2011; Chown et al. 2004; Gunderson and Stillman 2015). Recent studies that directly relate basal heat tolerance to habitat thermal maxima find the expected positive relationship for basal heat tolerance (Comte and Olden 2017b; Sunday et al. 2019), but only a few studies directly related plasticity in heat tolerance to thermal variability. These studies found no relationship for freshwater fish (Comte and Olden 2017b) or only a weak relationship for terrestrial taxa (Gunderson and Stillman 2015).

Here we draw on 3000+ data records of heat tolerance covering 500+ fish species from freshwater, brackish and marine habitats to test the hypothesis that variation in heat tolerance exhibited by species is related to thermal conditions to which they are exposed within their geographic distribution. Our study differs from previous work in three ways. First, we explicitly test whether latitude is an adequate proxy for thermal conditions, rather than assuming this relationship. Second, we disentangle thermal conditions into thermal extremes and temporal variation (seasonal fluctuations), thereby examining multiple dimensions of habitat temperature. Third, we leverage an unprecedented dataset of thermal tolerance data of fishes, spanning marine, freshwater, and brackish species, which allows us to compare thermal sensitivity and plasticity across realms. Due to their smaller volume, freshwater bodies (e.g., ponds, lagoons, lakes) have greater diurnal and seasonal temperature fluctuations, and exhibit more extreme temperature values compared to marine habitats. We therefore hypothesize that freshwater fish are most heat tolerant and exhibit the highest plasticity in heat tolerance.

We focus on fishes as they inhabit both marine and freshwater habitats globally, and are the most diverse vertebrate group, with 31,000+ species (Rabosky et al. 2018). The advantage of using a single taxonomic group is that it reduces the influence of possible confounding factors that may drive observed differences in heat tolerance and plasticity thereof. For example, many large‐scale comparisons of thermal tolerance document clear differences between terrestrial and marine systems, but such comparisons frequently involve aquatic and terrestrial organisms for which different methodologies may be employed and that differ in other respects, such as mode of breathing and evolutionary history, which are known to affect heat tolerance (Bennett et al. 2021; Leiva et al. 2019; Rezende et al. 2014; Verberk et al. 2016).

We capitalize on the advent of global water temperature models (Barbarossa et al. 2021; Wanders et al. 2019) and datasets (e.g., NOAA Global Surface Temperature Dataset) to characterize the thermal regimes of aquatic habitats, which are not well represented by air temperature measurements. Using these global datasets on thermal regimes in combination with our novel fish thermal tolerance dataset, we (i) test the extent to which the thermal tolerance of fishes and its plasticity are related to the thermal regimes of their habitats, (ii) estimate species' climate vulnerability based on the difference between heat tolerance and the maximum habitat temperature (Comte and Olden 2017a; Deutsch et al. 2008), and (iii) evaluate the extent to which plasticity in heat tolerance may reduce climate vulnerability through time gained for evolutionary adaptation (Morley et al. 2019).

## 2 Methods

### 2.1 Thermal Tolerance Data Collection

We compiled thermal tolerance data combining three existing databases: GlobTherm, which is a global database of experimentally derived thermal tolerance values across various species groups (Bennett et al. 2018), and the databases from two other thermal tolerance studies (Comte and Olden 2017a; Leiva et al. 2019). From these databases, we selected records concerning fishes, removed duplicates and then supplemented these data through an additional literature search for more recent studies (from 2015 onwards), using the Web of Science search engine and a combination of the search terms ‘fish’ and ‘ctmax’, ‘critical thermal maximum’, ‘thermal limit’ or ‘thermal tolerance’. From the search result, we selected papers that report on the thermal tolerance of fish species expressed as the critical thermal maximum (CT) or lethal thermal maximum (LT), obtained in dynamic and static assays, respectively. In addition to the thermal thresholds, we also extracted relevant information on the experimental set‐up, namely the ramping rate, trial duration, starting temperature, acclimation temperature and acclimation duration. We also obtained the latitude from which the tested fish originated. The three existing databases supplemented with the results of the literature search yielded 3257 upper thermal tolerance records from 569 species (Verberk et al. 2026). We classified each species into one of three groups based on information on realm (freshwater, brackish, marine) and migration obtained from FishBase and targeted internet searches: (1) fish predominantly using freshwater, including catadromic fish which grow up in freshwater habitats, such as Eel ( *Anguilla anguilla* ), (2) fish predominantly using marine waters, including anadromic fish which grow up in the ocean, such as salmonids and (3) brackish fish, including amphidromic fish. This classification resulted in 298 freshwater species, 209 marine species and 62 brackish water species.

### 2.2 Thermal Tolerance Data Harmonisation

Upper thermal limits of fish are quantified with different protocols. Static or LT assays expose fish to a constant temperature and note mortality after a set amount of time. The lethal temperature expresses the temperature at which the animals succumb to heat stress after the set amount of time (typically 50% mortality, but sometimes 100%). In dynamic or CT assays, the temperature that fish are exposed to is gradually ramped up and the temperature at which animals succumb is noted. However, tolerance to heat stress, like any other stressor, depends on both the stress intensity and its duration (Rezende et al. 2014). As the duration of dynamic assays varies not only because of physiological differences (more heat tolerant individuals take longer to succumb to heat stress), but also on methodological variables (starting temperature and ramping rate), thermal thresholds recorded in CT assays are not directly comparable. Accounting for differences in duration helps towards making thermal thresholds comparable across studies and species (Leiva et al. 2019; Molina et al. 2025). To isolate the contribution of methodology to assay duration, we bootstrapped the data for dynamic assays 50 times, taking only a single CT value for a given species and study. Next, we combined the subset with the data from static assays and fitted a model that included the duration of the assay (in hours; log‐transformed) as a fixed variable. This revealed that heat tolerance is negatively related to exposure duration (Figure S1), confirming previous work (Leiva et al. 2019; Rezende et al. 2014; Verberk et al. 2023). We then used the fitted slope estimate (−1.26 ± 0.0147 [SD]) to standardise all the reported heat tolerances to a duration of 1 h. Note that our value agrees well with a recent study by Molina et al. (2025) which takes a similar approach. Raw values and standardised values were tightly correlated (Figure S1), but even so, after the standardisation, we no longer find a significant difference in the reported heat tolerance values between static and dynamic assays across all data (*p* = 0.213), suggesting that differences in heat tolerance between both types of assays are largely driven by differences in duration of exposure.

### 2.3 Thermal Exposure

For each fish species, we characterised the thermal regime it is exposed to based on two key characteristics: the maximum weekly water temperature and the variability in weekly water temperature within its geographic range. To that end, we obtained geographic range maps of the species from AquaMaps (https://www.aquamaps.org; Kaschner et al. 2019) or, if unavailable, from the International Union for the Conservation of Nature (https://www.iucnredlist.org; IUCN 2024). Next, we overlaid these maps with water temperature data. For freshwater fishes, we used weekly water temperature data obtained from FutureStreams at a 5 arcmin spatial resolution (Bosmans et al. 2022). Similar to Barbarossa et al. (2021), we took the maximum weekly temperature of each year, averaged this over a 30‐year climatology period (1976–2005), and selected, per species, the 97.5 percentile across the range as the maximum habitat temperature. To determine temporal variability in temperatures experienced, we quantified the within‐year coefficient of variation of the weekly temperature. To do so, we first quantified per gridcell the coefficient of variation across the weeks for each year, averaged these coefficients of variation across years, and took the mean across the gridcells within the range of each species, thus arriving at a single value for each species. For marine and brackish fish species, we took a similar approach using weekly data on sea surface temperature from the NOAA OI SST V2 High Resolution dataset (years 1982–2011), with a spatial resolution of 15 arcmin (https://psl.noaa.gov/; Huang et al. 2021).

Next, we tested for differences in thermal exposure between marine, brackish and freshwater fishes based on the 523 fish species for which we had data on both latitude (*n* = 550) and thermal regime (i.e., maximum habitat temperature and habitat thermal variability) (*n* = 538). Since we had only a single value for maximum habitat temperature and thermal variability per species (derived from its geographic distribution, see above), we also subsetted the data so that we had a single value for latitude, selecting the record with median latitude when we had data spanning different latitudes. Next, we used Phylogenetic Generalised Least Squares (PGLS) regression to account for potential phylogenetic non‐independence due to shared evolutionary history between species (Figure S2). We constructed separate models for maximum habitat temperature and habitat thermal variability and in each model, we included realm (freshwater, brackish, marine) and absolute latitude, with latitude as a second order polynomial to account for non‐linear relationships.

### 2.4 Thermal Sensitivity

To evaluate how thermal exposure may affect fish heat tolerance we fitted three models. The first two models are used to compare how well variation in heat tolerance can be explained by either thermal conditions (thermal extremes and seasonal fluctuations in temperature) or latitude (as a proxy for thermal conditions). These models included heat tolerance (corrected for trial duration, see above) as a dependent factor and included as explanatory variables either latitude (model 1) or thermal conditions (thermal maxima and thermal variability, model 2). We also included acclimation temperature and the interaction between acclimation temperature and either latitude or thermal variability to test whether species from experiencing more variable thermal conditions (or living at latitudes characterised by more variable temperatures) also exhibited greater plasticity. In addition, we included a second order orthogonal polynomial for acclimation temperature in all models to account for increasingly smaller increases in heat tolerance with increasing acclimation temperatures. Preliminary analysis showed that models including this second order polynomial for acclimation temperature were better supported than models without. To allow for both non‐linear responses to latitude and different latitudinal responses across hemispheres, we included latitude and the second and third order polynomial.

Model 1:

$$\begin{array}{c} \text{Heat tolerance}\sim \mathrm{Lat}+{\mathrm{Lat}}^{2}+{\mathrm{Lat}}^{3}+\text{AccT}+\text{AccT}:\mathrm{Lat}+\text{AccT}:{\mathrm{Lat}}^{2}\\ +\text{AccT}:{\mathrm{Lat}}^{3}+{\text{AccT}}^{2} \end{array}$$

Model 2:

$$\begin{array}{c} \text{Heat tolerance}\sim \text{MaxHabitatT}+\text{Tvariability}+\text{AccT}\\ +\text{AccT}:\text{Tvariability}+{\text{AccT}}^{2} \end{array}$$

To account for multiple records per species, we used a Bayesian phylogenetic multilevel modelling framework, which can include random effects for both phylogeny (Figure S2) and species identity (Bürkner 2017). For models 1 and 2, we included 510 species for which we had data on acclimation temperature, latitude, and habitat thermal regime. We compared the two models by calculating the marginal and conditional *R* ^2^ values. We also used the second model to test whether species exposed to hotter conditions are more heat tolerant and whether species experiencing greater seasonal variation in habitat temperature are more plastic. Next, we fitted a third model to test how heat tolerance and its plasticity vary between fishes from freshwater, brackish, and marine habitats.

Model 3:

$$\begin{array}{c} \text{Heat tolerance}\sim \text{MaxHabitatT}+\text{Tvariability}+\text{AccT}\\ +\text{AccT}:\text{Tvariability}+\text{Realm}+\text{AccT}:\text{Realm}+{\text{AccT}}^{2} \end{array}$$

This model was similar in structure to model 2, but in addition also included realm and the interaction between realm and acclimation temperature to test for differences in plasticity in heat tolerance between fishes from freshwater, marine and brackish habitats. Since this model did not include latitude, we could include species for which latitude was missing but other variables were available, bringing the number of species in this model to 525.

Correlation plots (Figure S3) revealed that the actual acclimation temperature (i.e., the non‐standardised values) was strongly correlated with maximum habitat temperature (tropical fishes are typically acclimated to higher temperatures than polar fishes). We therefore standardised acclimation temperature relative to the mean acclimation temperatures for a given species (acclimation temperature—mean acclimation temperature). Standardised acclimation temperatures were no longer correlated with either latitude, maximum habitat temperature or habitat thermal variability (Pearson R, *p* > 0.102), increasing model reliability. The effect of (standardised) acclimation temperature expresses the change in heat tolerance per degree change in acclimation temperature, which is identical to the ARR. Hence, the interaction between thermal variability and acclimation temperature allows us to calculate how ARR values differ between thermally constant and thermally variable habitats. Similarly, the interaction between realm and acclimation temperature allows to estimate how ARR differs between species of different realms.

To check whether our measure of ARR was confounded by local adaptation, we performed a sensitivity analysis, re‐running model 2 with a random effect structure where we included not only species as a random intercept, but also added latitude of collection nested within species. This procedure fits random intercepts for each population within a species and thus controls for the confounding effect of local adaptation. In addition, we subsetted the data to only include studies with multiple acclimation temperatures for a given population and re‐ran model 2. The fitted estimates for ARR were very similar across models (original model: 0.20 [0.16–0.23]; model with random intercepts for population within a species: 0.22 [0.19–0.25]; model on subsetted data: 0.22 [0.19–0.25]). Such small differences can be explained by the structure of the underlying data: the dataset includes a wide temperature range across acclimation conditions, but only a narrow latitudinal range across populations (Table 1). Given the reported effect of differences in latitude on heat tolerance due to local adaptation (Sasaki et al. 2022) and that of acclimation temperatures due to physiological plasticity (Morley et al. 2019), we would expect the effects of acclimation temperature to be an order of magnitude greater than those of local adaptation of populations at different latitudes. This is confirmed by our sensitivity analysis showing that the value of ARR remained very similar irrespective of whether the effects of local adaptation were accounted for or not, indicating that ARR values reflect the effect of phenotypic plasticity, rather than local adaptation. Given the negligible differences, we proceeded with the simpler random effect structure.

**TABLE 1:** Attributes of the full dataset used in this study.

Dataset attribute	All data	Marine	Freshwater	Brackish
Records	3257	1036	1860	361
Species	569	209	298	62
Populations (i.e., species records at different latitudes)	1001	324	585	92
% species with data from multiple populations	36.0% (205/569)	32.1% (67/209)	39.9% (119/298)	30.6% (19/62)
Mean number of acclimation temperatures per species	3.04	2.61	3.37	2.89
% species with data from multiple acclimation temperatures	57.5% (327/569)	50.2% (105/209)	63.8% (190/298)	51.6% (32/62)
Mean range in acclimation temperatures	7.52°C	5.62°C	8.98°C	6.52°C
Mean range in latitude	1.26 degrees	1.23 degrees	1.36 degrees	0.95 degrees

### 2.5 Predicting Plasticity in Heat Tolerance Globally

We used our third model to predict global patterns in vulnerability to overheating, using global maps of maximum weekly water temperature and thermal variability for each gridcell as input. We predicted warming tolerance (in degrees °C) as the difference between (modelled) heat tolerance and maximum habitat temperatures (Deutsch et al. 2008). We also predicted the additional time (as a %) that is provided by acclimation until overheating occurs, which is calculated by comparing the time t_1_ until overheating without plasticity (i.e., ARR = 0) and the time t_2_ with plasticity (see Figure S6 for a conceptual explanation).

(1) $$t_{1}=\frac{\text{Maximum}\;{\text{habitat temp}}_{\text{gridcell}}-{\text{heat tolerance}}_{\text{gridcell}}}{\text{rate of warming}}$$

(2) $$t_{2}=\frac{\text{Maximum}\;{\text{habitat temp}}_{\text{gridcell}}-{\text{heat tolerance}}_{\text{gridcell}}}{\text{rate of warming}}\times \frac{1}{1-{\mathrm{ARR}}_{\text{gridcell}}}$$

When calculating the relative difference between *t*_1_ and *t*_2_, this additional time gained (%) is independent from the rate of warming (faster rates lead to proportional reductions in both *t*_1_ and *t*_2_) and can be simplified as:

(3) $$t_{3}=\left(\frac{\;t_{1}}{t_{2}}-1\right)\times 100\%=\left(\frac{1}{1-{\mathrm{ARR}}_{\text{gridcell}}}-1\;\right)\times 100\%$$

To predict heat tolerance for each gridcell, we used model 3, using the maximum weekly temperature and thermal variability of each gridcell as input layer and calculating this separately for freshwater temperature maps and sea surface temperature maps. Another factor influencing predictions of heat tolerance and its plasticity is the acclimation temperature itself, since models with a quadratic effect for acclimation temperature were better supported. Thus, plasticity in heat tolerance is progressively reduced at higher actual acclimation temperatures. Since the spatial predictions are not based on any particular species, the acclimation temperature that we deemed ecologically relevant was the value that coincides with the maximum weekly water temperature in each gridcell, which was derived from the empirical relationship between maximum habitat temperatures and actual acclimation temperatures (Figure S4; points above the red line indicate positive standardised values, those below indicate negative values). Second, we used model 3 to predict heat tolerance at both the standardised acclimation temperature and at a standardised acclimation temperature of +1°C (which could be seen as a warming scenario); the difference in model predictions represents the ARR. Third, we combined results from freshwater and marine temperature maps to generate global, cross‐realm predictions of warming tolerance and time gained by acclimation.

## 3 Results

### 3.1 Thermal Exposure

**FIGURE 1:**
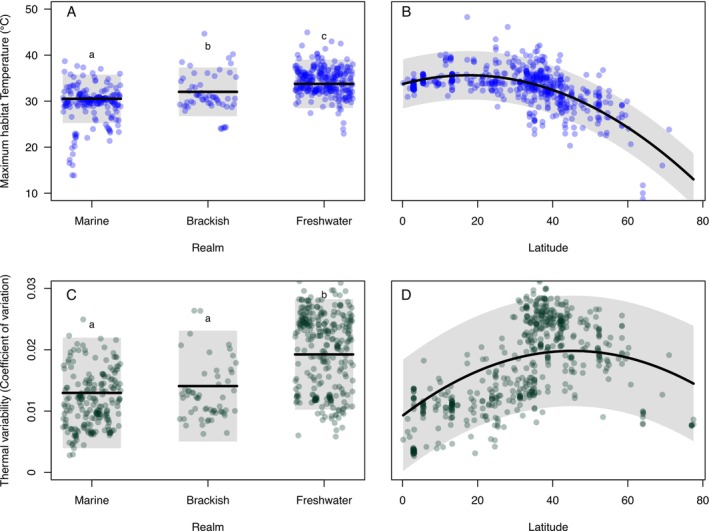
Partial residual plots for maximum habitat temperature (A, B), and thermal variability (C, D) for the 523 species of fishes for which we had data on latitude and thermal conditions in their distribution range. Each point represents the thermal conditions for a single species. A and C show differences between fishes from marine, brackish and freshwater habitats, while B and D show the latitudinal relationships.

Freshwater fishes experience both higher maximum temperatures and higher thermal variability in their geographic range than marine species (Figure 1; PGLS: *t*_1,518_ > 6.68, *p* < 0.001). Brackish species also inhabit warmer habitats than marine fishes (PGLS: *t*_1,518_ = 2.94; *p* = 0.0034), but the thermal variability in their geographic range does not exceed that of marine fishes (PGLS: *t*_1,518_ = 1.68; *p* = 0.093). At the median latitude, the maximum water temperature that freshwater fishes experience in their habitats is 3.23°C higher than that of marine fishes (Figure 1A), while the thermal variability is 0.0063 (48%) higher (Figure 1C). Both maximum habitat temperatures and the thermal variability associated with the geographic range of a species are strongly phylogenetically structured for both maximum habitat temperatures and thermal variability (lambda values of 0.87 and 0.93, respectively). Both thermal exposure metrics were strongly correlated with absolute latitude: thermal maxima were highest in the tropics (Figure 1B), while thermal variability peaked at temperate latitudes (Figure 1D).

### 3.2 Thermal Sensitivity

**FIGURE 2:**
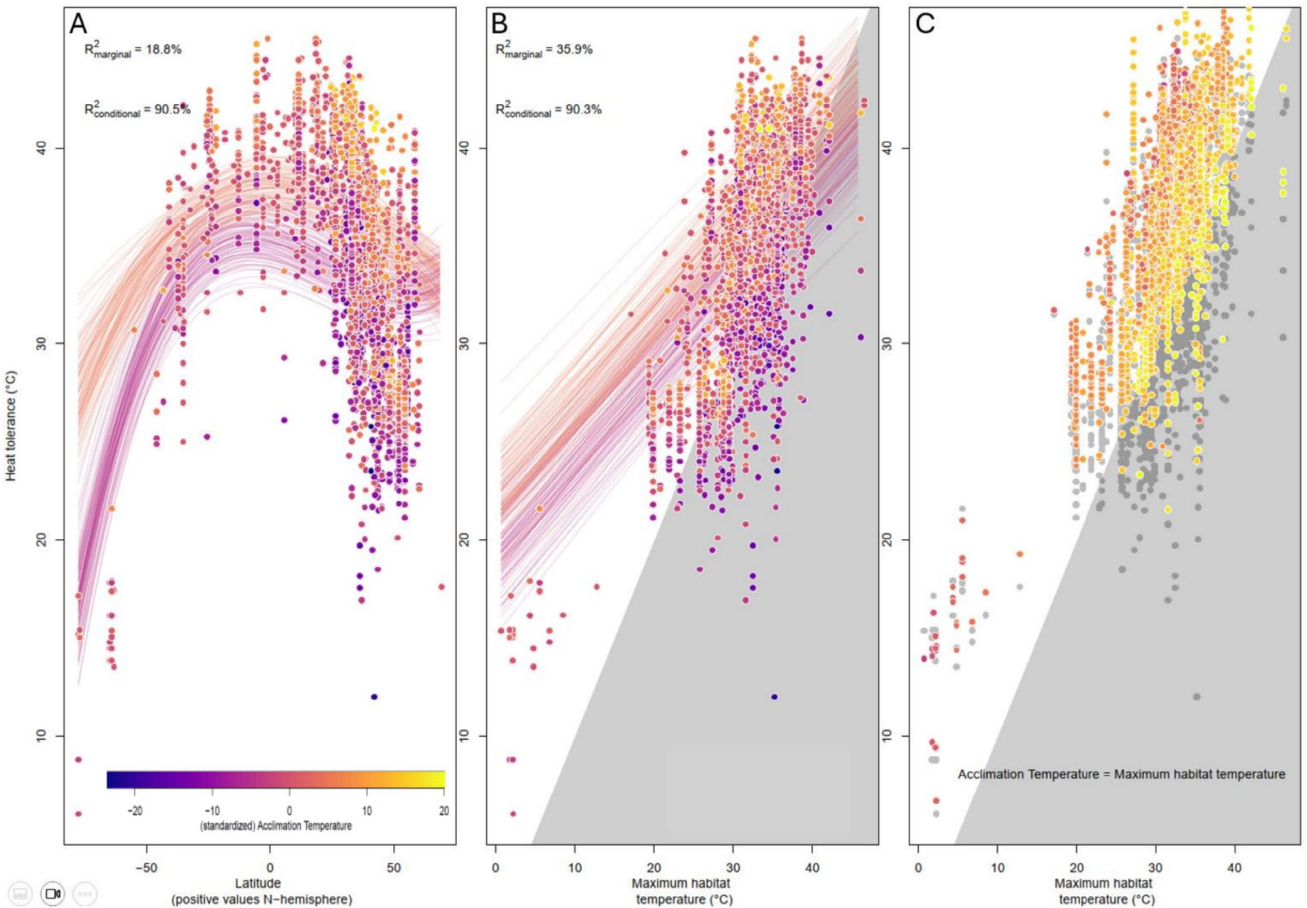
Heat tolerance in relation to latitude (panel A), and maximum habitat temperature (panels B and C). This plot includes multiple data points (*n* = 2743) comprising 510 species. Data points represent separate measurements and are colour‐coded for different acclimation temperatures, with blue/purple corresponding to lower acclimation temperatures and red/orange to higher acclimation temperatures. Lines in panels A and B show the model posterior predictions for two different standardised acclimation temperatures (−5 and +5). The grey polygon in panel B highlights when observed heat tolerance falls below maximum habitat temperatures. In panel C, predicted heat tolerance values are shown in colour, assuming the species is acclimated to the standardised acclimation temperature that corresponds to the maximum habitat temperature (Figure S4), while grey points indicate raw data.

Heat tolerance of fishes varied strongly with both latitude (Figure 2A; Table S1) and thermal conditions experienced by fish in their habitat (Figure 2B; Table S2) In line with previous work, heat tolerance was highest in tropical fishes and declined away from the equator. However, the effect of latitude differed between the northern and southern hemispheres, which could reflect asymmetry between the two hemispheres in latitudinal variation in temperature and its variability (Chown et al. 2004). In addition, for temperate regions in the northern hemisphere (35–66.5 N), there was a large variation in heat tolerance despite a limited latitudinal range. Patterns in heat tolerance were better captured by a model based on the thermal habitat conditions (marginal *R* ^2^ = 35.9%), compared to a model based on latitude (marginal *R* ^2^ = 18.8%). The random factors phylogeny and species identity also explained a large part of the variation, resulting in total explained variation of around 90% (conditional *R* ^2^) for both models.

Paradoxically, 29% of the data records indicated fish experiencing maximum water temperatures in their habitat that exceeded their reported heat tolerance, resulting in a negative warming tolerance (i.e., heat tolerance minus maximum habitat temperature, points in the grey triangle in panel 2B). Negative warming tolerance values may arise when heat tolerance is measured in fish that are acclimated to temperatures below the maximum habitat temperature, resulting in heat tolerance values that underestimate the maximum heat tolerance of that species. In our dataset, actual acclimation temperatures increased with maximum habitat temperatures, that is, fishes from warmer habitats tend to be acclimated to temperatures further away from their maximum habitat temperature (Figure S4). With our second model, we used this information to predict the heat tolerance if that species would be acclimated to its maximum habitat temperature. The majority of these predicted heat tolerance values (i.e., 93%) were above the maximum habitat temperature (Figure 2C), resulting in positive warming tolerances.

**FIGURE 3:**
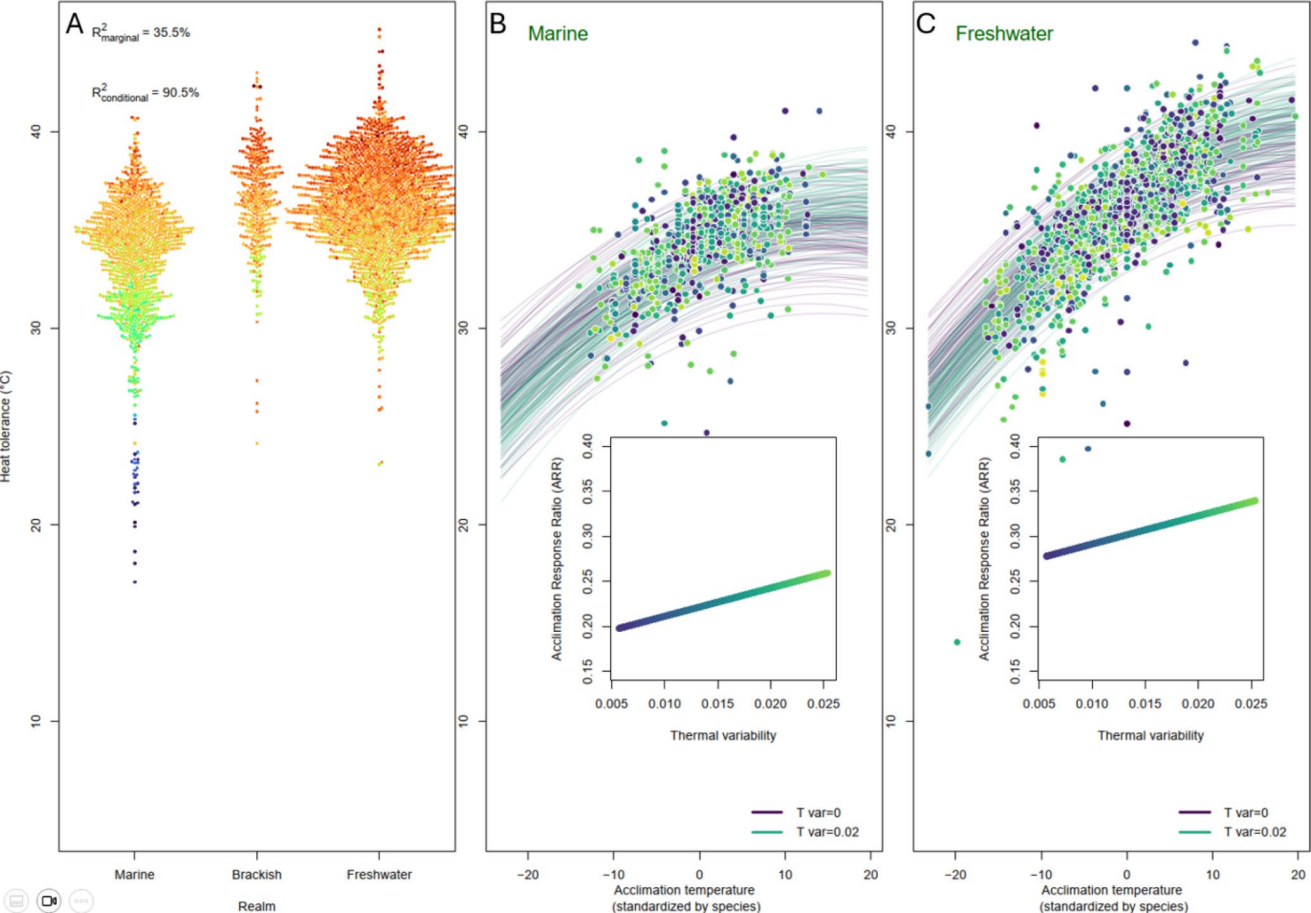
Heat tolerance for fishes from marine, freshwater, or brackish habitats (panel A). This plot includes multiple data points (*n* = 3134) comprising 525 species. Data points represent separate measurements and are colour‐coded by maximum habitat temperature, with blue corresponding to low habitat temperatures and red corresponding to high habitat temperatures. Effects of acclimation temperature are shown separately for marine fishes (Panel B) and freshwater fishes (panel C). Lines in panels B and C show the model posterior predictions for thermally stable habitats (purple) and thermally variable habitats (green). Data points are also colour‐coded according to habitat thermal variability. The inset plot shows how the slope of the lines (i.e., the Acclimation Response Ratio, ARR, calculated when the standardised acclimation temperature is set to 0) varies with thermal variability, being lower in marine than in freshwater fishes.

Freshwater fishes had higher reported heat tolerance than marine species (Figure 3A) and this difference reflected their exposure to higher water temperatures (Figure 1A). Plasticity in heat tolerance also varied across species, with freshwater fishes and species from thermally variable habitats exhibiting greater plasticity than marine fishes and species from habitats with lower seasonality (Figure 3B,C). In model 3, interactions of (standardised) acclimation temperature with both realm and thermal variability were significant (Table S3), indicating independent contributions to explaining variation in plasticity. In other words, freshwater fishes are more plastic even when accounting for the greater thermal variability they experience (Figure 1C). In addition, we found a significant quadratic effect in all models such that gains in heat tolerance diminished with increasing acclimation temperature. Fishes from brackish waters resembled those from fresh waters in both their basal heat tolerance and their plasticity in heat tolerance (Table S3).

**FIGURE 4:**
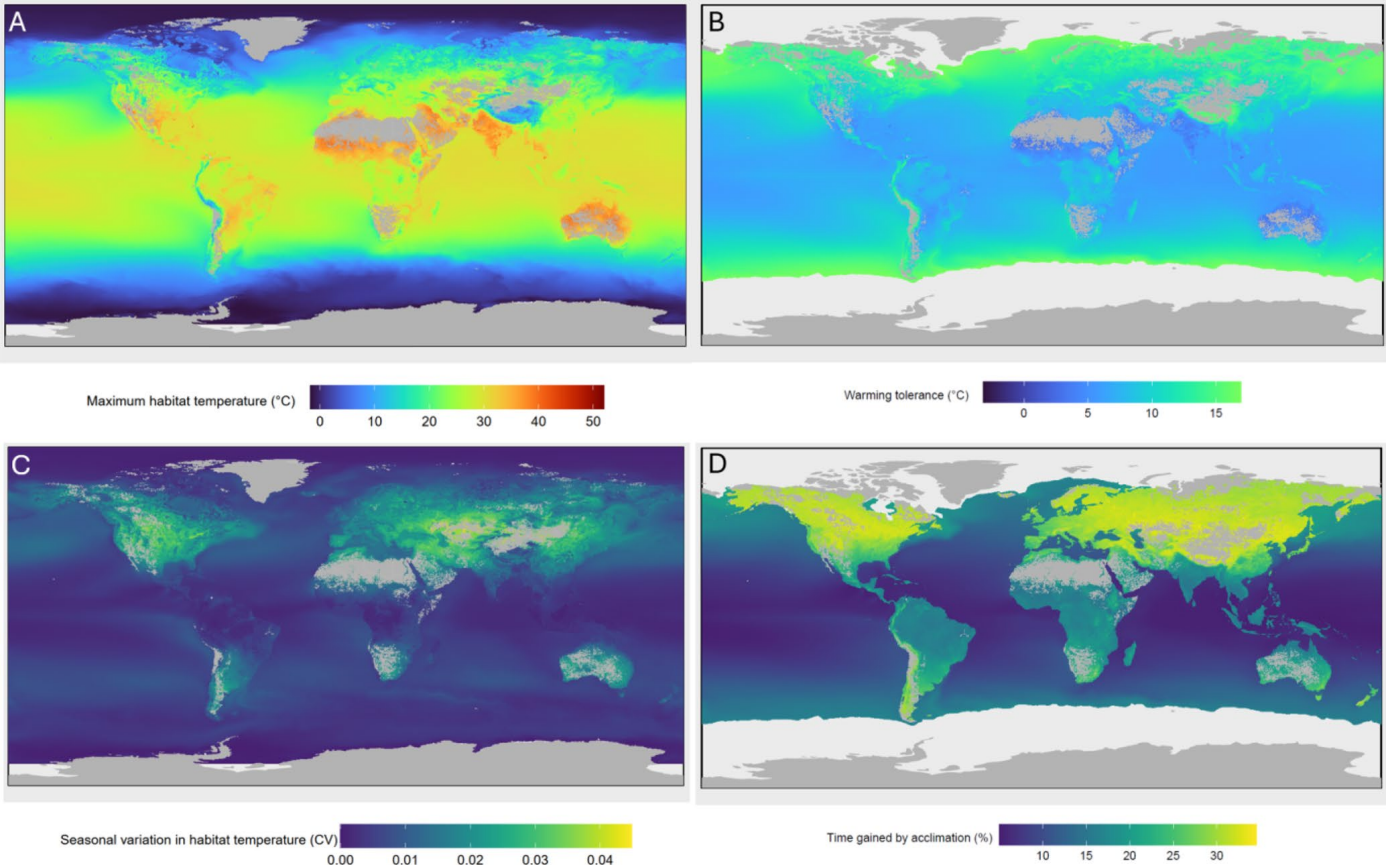
Global variation in maximum weekly habitat temperature (panel A), seasonal variation in habitat temperature (panel C), warming tolerance (panel B), which is the difference between heat tolerance and maximum habitat temperature, and benefits of plasticity, expressed in additional time gained for evolutionary adaptation by acclimation (%) (panel D). Colours in panels A and B range from cold (dark blue) to warm (dark red), while in panels C and D they range from thermally constant/short time gains (blue) to thermally variable/high time gains (yellow). Regions in plot B and D with high model uncertainty (see Figure S5) are without colour.

Our model enabled us to use habitat thermal extremes (Figure 4A) and seasonal variation in habitat temperature (Figure 4C) to predict heat tolerance (Figure 2C), which allows for a global prediction of warming tolerance (Figure 4B), that is, the difference between heat tolerance and the maximum habitat temperature. This application shows that tropical fishes live closer to their upper thermal limits while fishes in temperate and polar regions have higher warming tolerances, confirming earlier work (Comte and Olden 2017a). Given that benefits of acclimation are increasingly smaller at higher acclimation temperatures, fishes from warm, tropical latitudes not only have smaller warming tolerances, but benefit less from plasticity, with time gains from plasticity of ~5%. In contrast, temperate fishes, especially those in freshwaters, exhibit greatest plasticity, resulting in up to 35% more time until overheating (Figure 4D).

## 4 Discussion

To effectively use ecophysiological metrics in predicting vulnerability to climate warming, these metrics must be under selection, responding to the thermal conditions that species have encountered throughout their evolutionary history. We observed a strong phylogenetic structuring, both in terms of their exposure, that is, the thermal regime fishes experience in their habitat, and in their sensitivity, that is, the experimentally derived tolerance values. This finding aligns with previous work demonstrating a strong phylogenetic structuring of heat tolerance (Bennett et al. 2021; Leiva et al. 2019; Ruthsatz et al. 2024). Our study further demonstrates that latitudinal patterns in heat tolerance and its plasticity reflect geographic differences in maximum habitat temperature and habitat thermal variability (Figure 2). In principle, this result validates the use of latitude as a proxy (Pinsky et al. 2019), but we also show that spatial differences in thermal extremes and variability provide a superior explanation for distinct heat tolerance adaptations (Comte and Olden 2017b; Gunderson and Stillman 2015; Sunday et al. 2019).

Against the backdrop of a warming climate, studies focus increasingly on plasticity of heat tolerance—the ability of fishes and other organisms to acclimate to warmer conditions (Gunderson and Stillman 2015; Rohr et al. 2018; Weaving et al. 2022; Verberk et al. 2023). The acclimation response ratio (ARR) is a popular metric to express differences in plasticity. Given that many biological rates scale exponentially with temperature, there are clear upper ceilings to increasing heat tolerance following acclimation (Payne and Smith 2017; Sandblom et al. 2016; Stillman 2003). In our analysis we consistently found a curvilinear relationship between heat tolerance and acclimation temperature, with diminishing gains in heat tolerance with increasing acclimation temperature (Figure 2C). This likely reflects the challenges involved in physiologically adjusting to extreme heat stress, requiring adjustments across more and more processes (Ern et al. 2023; Sandblom et al. 2016). Since such diminishing gains are more prominent in warmer waters (Figure 4D), our results also provide support for a trade‐off such that species with a lower heat tolerance will have greater plasticity (Gunderson 2023).

There are likely evolutionary costs to maintaining plasticity (Comte and Olden 2017b). This likely explains why reported values for ARR are below 1 and even predominantly below 0.5 (Weaving et al. 2022). If there are costs to plasticity, species from thermally stable environments should exhibit lower plasticity and high levels of plasticity should be restricted to species living in thermally variable habitats. Our study provides support for this hypothesis, as we found greater levels of plasticity in fishes from thermally variable habitats (Figure 4). Previous studies have tested this idea, but found no (Comte and Olden 2017b) or limited (Ruthsatz et al. 2024) statistical support, likely due to a more limited sample size (82 and 93 fish species in their studies, respectively, versus 500+ species in our study). In addition, by including both marine and freshwater fishes we sampled a greater range in thermal variability and could also test for differences in plasticity between both realms (Figure 3). Our results show that even after accounting for the effect of thermal variability, freshwater fishes still exhibit greater levels of plasticity. This could be related to freshwater fish being exposed to greater fluctuations in levels of dissolved oxygen in their habitats (Andersen et al. 2017; Verberk and Bilton accepted). There is evidence for synergistic effects between hypoxia and heat with hypoxia reducing tolerance to heat (Eliason et al. 2011; Jutfelt et al. 2021; Rubalcaba et al. 2020) and warming reducing tolerance to low oxygen (Deutsch et al. 2020; Verberk et al. 2022). Given these synergistic effects, protective mechanisms for heat and hypoxia may be shared (cross‐tolerance) (Sinclair et al. 2013). Thus, the greater fluctuations in dissolved oxygen experienced by freshwater fishes could drive greater acclimatory capacity to safeguard oxygen uptake and via cross‐tolerance explain their greater plasticity in heat tolerance. Oxygen conditions may also fluctuate in coastal habitats and brackish habitats, especially those affected by eutrophication (Giomi et al. 2019). Although our data comprised relatively few species from brackish habitats (10.9%), they did display thermal sensitivities comparable to those of freshwater habitats, highlighting the need for further study. An additional, not mutually exclusive explanation relates to differences in the ability to exploit small‐scale spatial variation in temperatures. There are fewer barriers to dispersal in marine ecosystems, when compared to freshwaters (Barbarossa et al. 2020). Consequently, freshwater fishes may require a greater capacity for acclimation because even if cooler habitats are nearby (i.e., an upper reach of the river, or a deeper lake), these may not be accessible.

Heat tolerance increased with maximum habitat temperature with a slope less than one (0.46–0.47; Tables S2 and S3). Consequently, warming tolerance, that is, the difference between heat tolerance and habitat temperature (Deutsch et al. 2008), was smaller for fishes from warmer habitats (Figure 4A). This indicates a greater climate vulnerability of fishes from warm, tropical habitats (Comte and Olden 2017a). However, given that biological rates, such as energy metabolism, (initially) scale exponentially with temperature (Fry 1947; Clarke and Johnston 1999; Rubalcaba et al. 2020), a small change in temperature could have a similar effect for a tropical fish as a much larger temperature change for a polar fish (Payne and Smith 2017).

In several instances the maximum habitat temperature exceeded the heat tolerance level of fishes. This discrepancy is particularly puzzling given that habitat temperatures were calculated over considerably longer timescales (i.e., a week) than 1 h, which was the duration to which heat tolerance experiments were standardised. Given that lower levels of heat stress can be tolerated over longer timescales (Leiva et al. 2019; Peralta‐Maraver and Rezende 2020; Rezende et al. 2014; Verberk et al. 2023) (Figure S1), warming tolerances will likely be even lower. Recently, Molina et al. (2025) indeed report much smaller warming tolerances, when employing longer, monthly timescales. One way to explain these discrepancies is that the reported heat tolerance levels analysed here may not reflect those exhibited by local populations during summer in the warmest part of the species' range. Indeed, when using our model to predict a species' heat tolerance while adjusting the acclimation temperature to the maximum habitat temperature experienced by that species, we observed much fewer instances where habitat temperature exceeded (predicted) heat tolerance (Figure 2C). Thermal tolerance can vary seasonally, and geographically across populations (Eliason et al. 2011; McKenzie et al. 2021; Sasaki et al. 2022; Zanuzzo et al. 2019), meaning that plasticity in heat tolerance but also genetic variation in heat tolerance across populations can help explain our observations on negative warming tolerances. In addition, thermal refuges at depth, or cooler sections of a stream may shelter animals from heat such that maximum habitat temperature calculated is higher than what species may experience during heat waves.

Studies on heat tolerance frequently juxtapose marine species against terrestrial species, but marine and terrestrial species differ in many respects, making it challenging to relate differences in heat tolerance to differences in thermal regimes experienced (Pinsky et al. 2019). Freshwater species can help resolve this problem, as demonstrated in our study. By drawing on data from an unprecedented number of fish species, our study spanned a large range in thermal variability and thermal extremes. Although fish as a group have a rich evolutionary history, all the species incorporated are aquatic and rely at least partly on gills to extract oxygen from the water, so variation across species is unlikely to be confounded by major differences in breathing mode, which is known to affect heat tolerance (Leiva et al. 2019). By drawing directly on information on habitat thermal regimes rather than using latitude as a proxy, we uncovered novel relationships between plasticity in heat tolerance and thermal variability and habitat use, which allowed us to predict global patterns in warming tolerance and benefits of acclimatory capacity expressed in time gained in much detail, based on maps of habitat thermal maxima and variability (Figure 4, Figure S5).

We acknowledge several limitations of our study. First, we treated species as the unit of investigation, using species distributions to estimate their exposure to habitat temperatures and evaluating thermal sensitivity at the species level. This approach overlooks potential variation among populations within species, which may differ in their thermal tolerance due to local adaptation or standing genetic variation. We could not test the extent to which local adaptation can modulate climate vulnerability given limited data on different populations spanning large differences in latitude and climate. Second, our analyses are constrained by the spatial and temporal resolution of available water temperature data. We acknowledge that organisms may experience their environments at much finer scales, and the mismatch between these scales may obscure the role of local conditions. In particular, small‐scale heterogeneity such as thermal refugia cannot be captured at the resolution of our data, yet such microhabitats may be critical for persistence under climate warming. Third, although our dataset is extensive, gaps remain, especially for taxa from colder regions which are undersampled (Figure S5). Addressing these limitations will require future studies that integrate high‐resolution environmental data with population‐level physiological measurements, enabling explicit tests of geographic variation in thermal tolerance and the role of local refugia. Such work will provide a more nuanced understanding of how species and populations can respond to ongoing climate change.

Nothwithstanding the need for further refinements, our results underscore the importance of thermal physiology for predicting responses to climate change, and highlight that plasticity in heat tolerance is an important mechanism to cope with thermal extremes. Going beyond latitude as a proxy, we show that thermal exposure has selected for unique heat tolerance adaptations in freshwater and marine fishes, highlighting the need to account for habitat‐specific temperature variations in ecological research and conservation efforts.

## Author Contributions

All authors contributed to the conceptualization of the study. Collection and curation of data on heat tolerance was led by Erin Henry, with contributions from Félix P. Leiva and Wilco C.E.P. Verberk; Valerio Barbarossa and Erin Henry performed the computations to obtain data on habitat temperatures; Wilco C.E.P. Verberk led the data analysis with input from all co‐authors. Wilco C.E.P. Verberk wrote the first draft of the manuscript, and all authors contributed substantially to revisions.

## Funding

This work was supported by Aard‐en Levenswetenschappen, Nederlandse Organisatie voor Wetenschappelijk Onderzoek, Vidi.213.093.

## Supporting information


**Data S1:** ele70341‐sup‐0001‐Supinfo.docx.

## Data Availability

The data and code supporting the findings of this study are published (Verberk et al. 2026) and are openly available at: https://doi.org/10.5281/zenodo.18266037.
