# Supplementary material for: Heat Tolerance and Its Plasticity in Freshwater and Marine Fishes Reflect Exposure to Extremes and Seasonal Variation in Habitat Temperatures
Source: Ecol Lett. 2026 Mar 4;29(3):e70341. doi: 10.1111/ele.70341 (PMC12959617; doi:10.1111/ele.70341)
Supplement: Supplementary file 1 — Data S1: ele70341‐sup‐0001‐Supinfo.docx. [file ELE-29-0-s001.docx]

**Supplementary materials:**

**Table S1. Summary table model 1: effects of latitude on heat tolerance.**

**Random Effects**

| Parameter | Estimate | Est. Error | l-95% CI | u-95% CI | Rhat | Bulk ESS | Tail ESS |
| --- | --- | --- | --- | --- | --- | --- | --- |
| Species | 2.135 | 0.120 | 1.904 | 2.374 | 1.000 | 5939 | 9801 |
| Phylogeny | 8.727 | 1.075 | 6.726 | 10.929 | 1.001 | 3277 | 6363 |

**Fixed Effects**

| Parameter | Estimate | Est. Error | l-95% CI | u-95% CI | Rhat | Bulk ESS | Tail ESS | p (MAP) |
| --- | --- | --- | --- | --- | --- | --- | --- | --- |
| Intercept | 34.75 | 3.51 | 27.70 | 41.58 | 1.00 | 4850 | 8550 | < .001 |
| Acclimation Temperature (standardized; Tacc) | 0.30 | 0.01 | 0.28 | 0.31 | 1.00 | 37362 | 17228 | < .001 |
| Latitude (1st polynomial) | -7.99 | 5.23 | -18.20 | 2.34 | 1.00 | 26369 | 17913 | 0.311 |
| Latitude (2nd polynomial) | -62.03 | 5.20 | -72.05 | -51.71 | 1.00 | 18370 | 18332 | < .001 |
| Latitude (3rd polynomial) | 24.48 | 4.61 | 15.41 | 33.40 | 1.00 | 19135 | 17546 | < .001 |
| Tacc (2nd polynomial) | -16.45 | 2.04 | -20.40 | -12.39 | 1.00 | 52295 | 16558 | < .001 |
| Tacc : latitude (1st polynomial) | -1.22 | 0.46 | -2.12 | -0.33 | 1.00 | 34503 | 17414 | 0.032 |
| Tacc : latitude (2nd polynomial) | 0.41 | 0.51 | -0.59 | 1.42 | 1.00 | 32890 | 17791 | 0.732 |
| Tacc : latitude (3rd polynomial) | -3.34 | 0.46 | -4.25 | -2.46 | 1.00 | 33659 | 19395 | < .001 |

**Table S2: Summary table model 2: effects of habitat thermal conditions on heat tolerance.**

**Random Effects**

| Parameter | Estimate | Est. Error | l-95% CI | u-95% CI | Rhat | Bulk ESS | Tail ESS |
| --- | --- | --- | --- | --- | --- | --- | --- |
| Species | 2.20 | 0.12 | 1.97 | 2.44 | 1.00 | 6257 | 11341 |
| Phylogeny | 7.16 | 0.98 | 5.35 | 9.15 | 1.00 | 3351 | 7113 |

**Fixed Effects**

| Parameter | Estimate | Est. Error | l-95% CI | u-95% CI | Rhat | Bulk ESS | Tail ESS | p (MAP) |
| --- | --- | --- | --- | --- | --- | --- | --- | --- |
| Intercept | 20.79 | 3.05 | 14.89 | 26.80 | 1.00 | 6740 | 11443 | < .001 |
| Acclimation Temperature (standardized; Tacc) | 0.20 | 0.018 | 0.16 | 0.23 | 1.00 | 37814 | 16119 | < .001 |
| Thermal variability of habitat temperature (Thab_var) | -18.03 | 9.42 | -36.43 | 0.54 | 1.00 | 41510 | 17209 | 0.160 |
| Maximum habitat temperatures (Thab_max) | 0.47 | 0.03 | 0.40 | 0.53 | 1.00 | 12106 | 16166 | < .001 |
| Tacc (2nd polynomial) | -16.15 | 2.07 | -20.22 | -12.09 | 1.00 | 59562 | 15936 | < .001 |
| Tacc : Thab_var | 5.24 | 0.87 | 3.53 | 6.97 | 1.00 | 37892 | 15505 | < .001 |

**Table S3. Summary table model 2: effects of habitat thermal conditions and realm on heat tolerance.**

**Random Effects**

| Parameter | Estimate | Est. Error | l-95% CI | u-95% CI | Rhat | Bulk ESS | Tail ESS |
| --- | --- | --- | --- | --- | --- | --- | --- |
| Species | 2.22 | 0.12 | 2.00 | 2.46 | 1.00 | 5431 | 10277 |
| Phylogeny | 7.00 | 0.96 | 5.21 | 8.97 | 1.00 | 3122 | 6911 |

**Fixed Effects**

| Parameter | Estimate | Est. Error | l-95% CI | u-95% CI | Rhat | Bulk ESS | Tail ESS | p (MAP) |
| --- | --- | --- | --- | --- | --- | --- | --- | --- |
| Intercept (Marine) | 20.63 | 3.00 | 14.72 | 26.54 | 1.00 | 5976 | 9654 | < .001 |
| Acclimation Temperature (standardized; Tacc) | 0.18 | 0.02 | 0.15 | 0.22 | 1.00 | 33907 | 17849 | < .001 |
| Tacc (2nd polynomial) | -16.59 | 2.01 | -20.52 | -12.68 | 1.00 | 52942 | 15951 | < .001 |
| Maximum habitat temperatures (Thab_max) | 0.46 | 0.03 | 0.40 | 0.59 | 1.00 | 10337 | 13926 | < .001 |
| Brackish | 0.79 | 0.49 | -0.17 | 1.74 | 1.00 | 13220 | 14883 | 0.266 |
| Freshwater | 0.076 | 0.46 | -0.84 | 0.98 | 1.00 | 12043 | 14561 | 0.973 |
| Tacc : Brackish | 0.080 | 0.019 | 0.04 | 0.12 | 1.00 | 41367 | 17452 | < .001 |
| Tacc : Freshwater | 0.081 | 0.014 | 0.05 | 0.11 | 1.00 | 37553 | 16345 | < .001 |
| Tacc : Thab_var | 3.125 | 0.995 | 1.15 | 5.07 | 1.00 | 32996 | 16667 | 0.0067 |

Figure S1


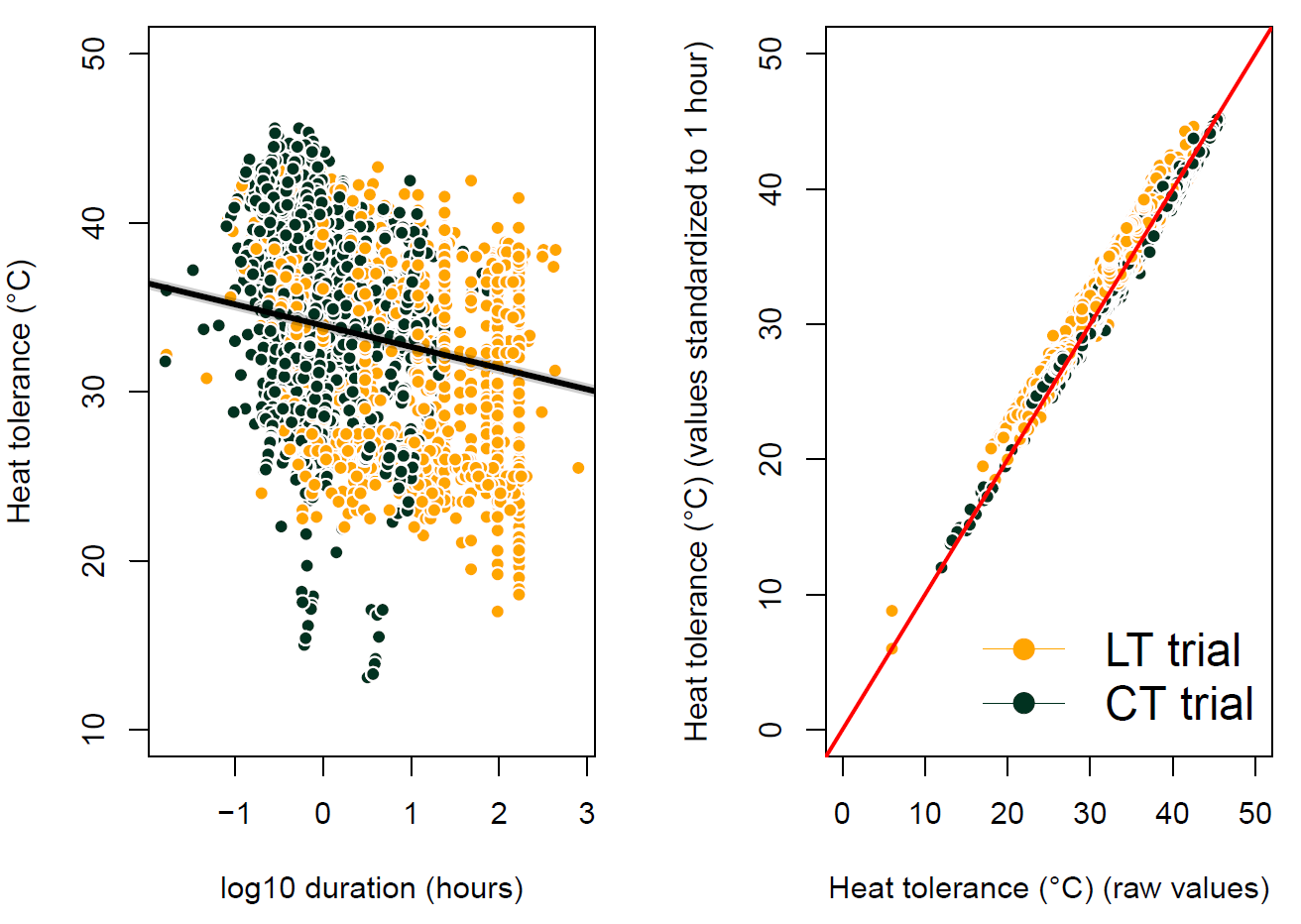


Figure S1: Effect of the duration of experimental assays to measure heat tolerance on the observed critical thermal maxima. Static assays (LT) typically have a longer duration than dynamic assays (CT) (left panel). Heat tolerance values before and after standardizing to a duration of 1 hour (using the negative correlation between heat tolerance and assay duration from the left panel), where tightly correlated (R^2^=0.96; right panel).

Figure S2


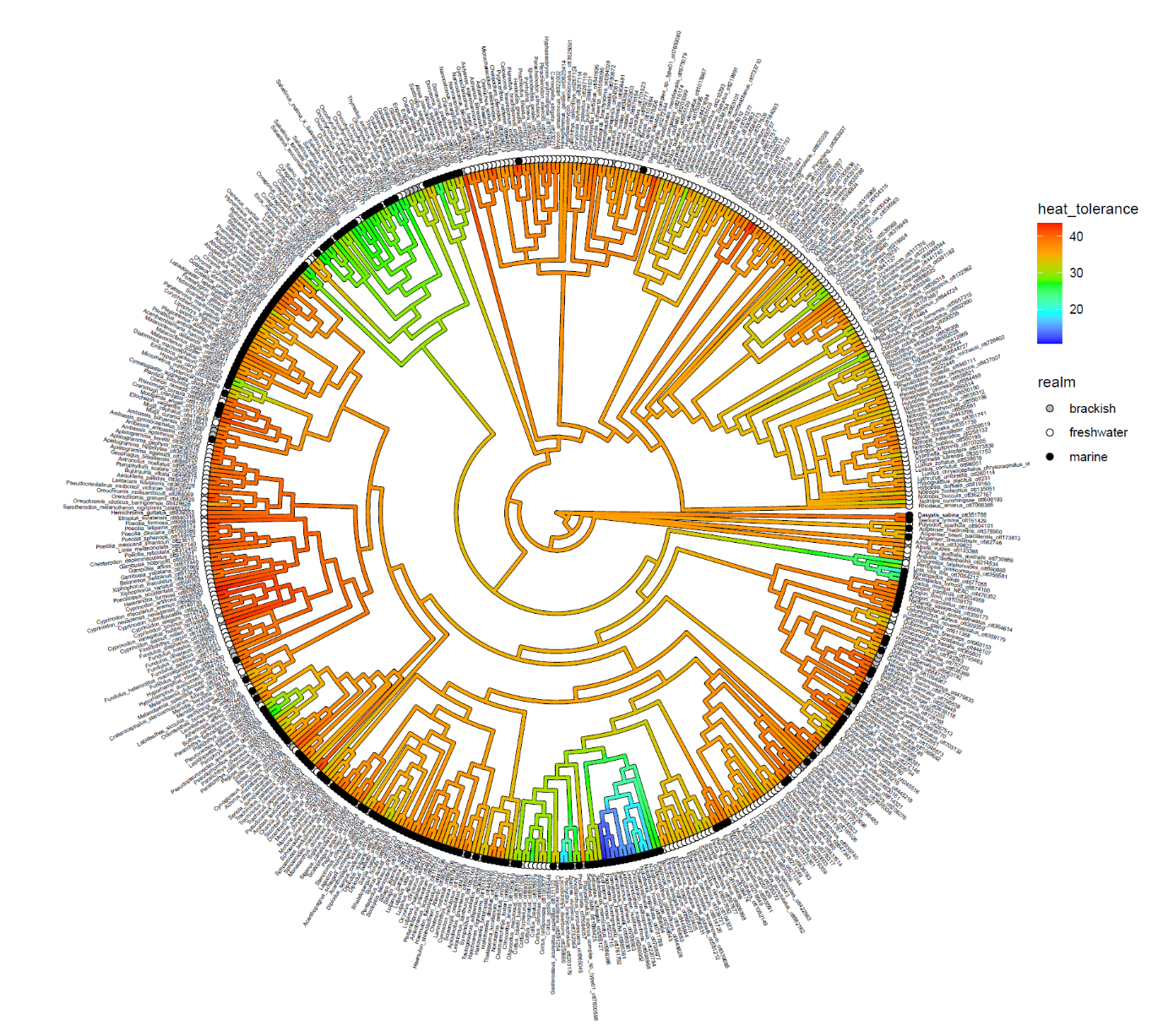
 Fig S2: Phylogeny of the species used in this study, branches and names are colour coded by their observed heat tolerance.

Figure S3


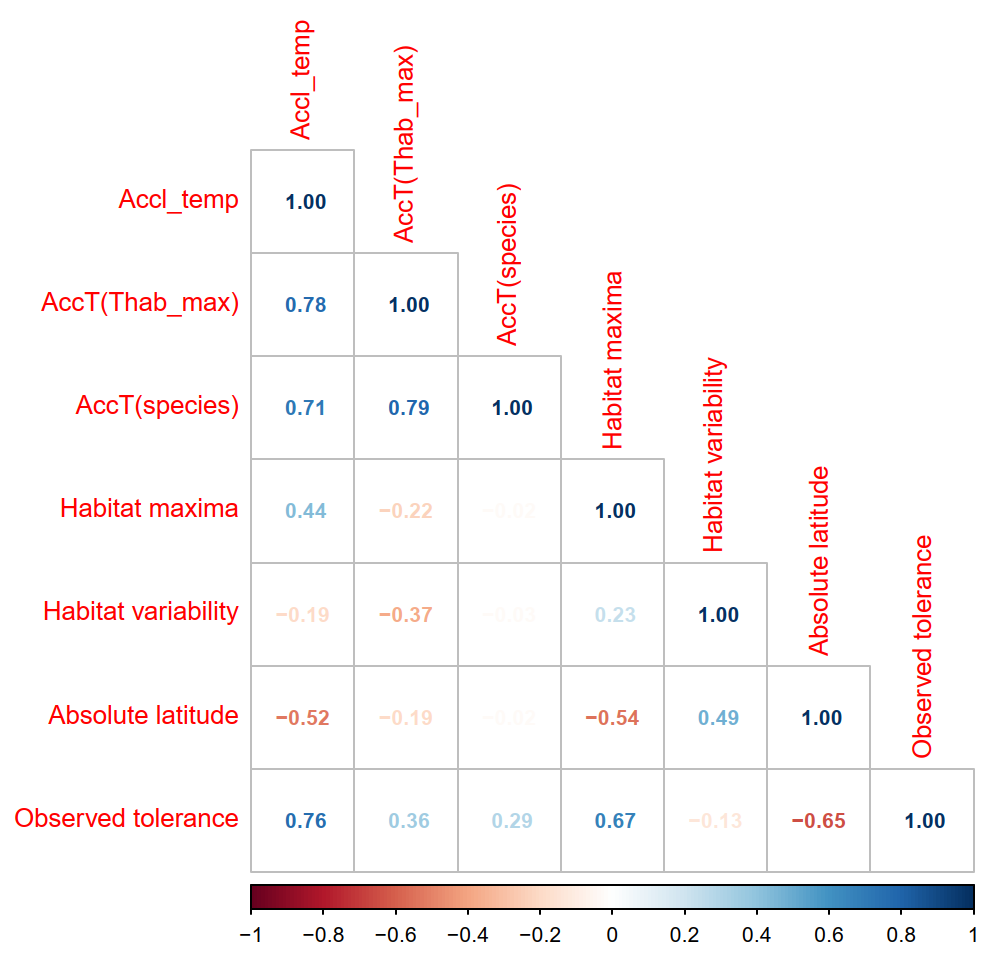


Figure S3: Correlation plot between the methodological parameter acclimation temperature (raw values and standardized by habitat temperature or species), environmental parameters (maximum habitat temperatures and thermal variability), latitude (presumed to be a proxy for these environmental parameters) and the heat tolerance observed in experimental assays.

Figure S4


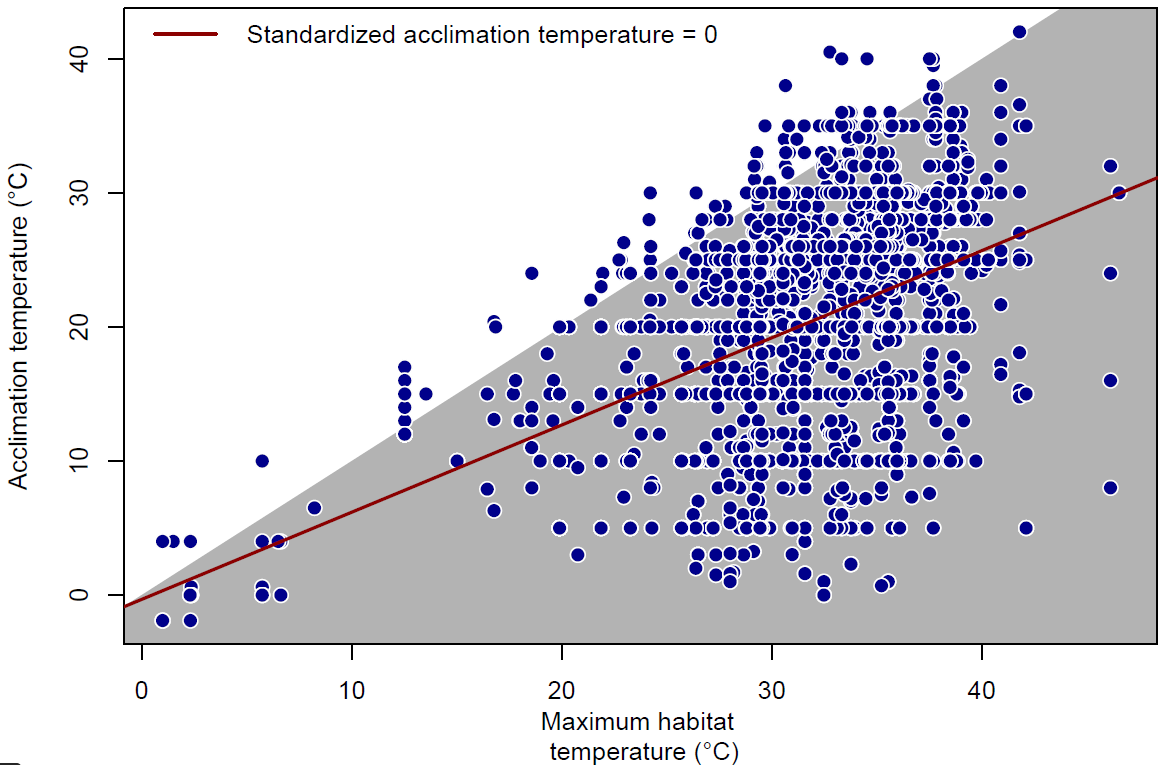


Fig S4. Acclimation temperatures plotted against maximum habitat temperatures. Species are rarely acclimated at temperatures above maximum habitat temperatures. We standardized acclimation temperatures per species by substracting the average acclimation temperature of all records for that species. The red line shows where on average the standardized temperature equals zero. While the standardized acclimation temperature increased with maximum habitat temperature, fishes from warmer habitats tend to be acclimated to temperatures further away from the maximum habitat temperature.

Figure S5

Fig S5. Global variation in plasticity of heat tolerance (ARR) predicted from our model, based on global maps of thermal variability and incorporating plasticity differences between marine and freshwater fishes. In addition, locations for all data records are plotted to display the geographic coverage of our data (A). Additionally, we plotted regions where model uncertainty was higher due to limited data (B). We did this by predicting heat tolerance for each combination of habitat temperature and seasonal variation using all the posterior draws (n=22,000) in model 3. Next, we calculated the difference between the 10th and 90th percentile as a measure of model uncertainty for all combinations of habitat temperature and seasonal variation. Regions where the model uncertainty exceeded the 75th percentile based on all possible combinations (i.e. large divergence between the 10th and 90th percentile of the predictions, so a high model uncertainty) are highlighted in black.

.

Figure S6

Fig S6. Conceptual explanation of how acclimation results in time gained before fish overheat. Time gained is calculated by comparing the time at which overheating occurs when there would be no plasticity (solid purple line) with the time until overheating when heat tolerance improves due to acclimation effects. To illustrate that the same perceptual time is gained irrespective of rate of warming, we show the time gain for slow rate of warming (a) and fast rate of warming (b).
